# Radiology imaging management in an Italian cancer center (IRST IRCCS) during the COVID-19 pandemic

**DOI:** 10.1186/s13244-020-00935-x

**Published:** 2020-12-03

**Authors:** Alice Rossi, Andrea Prochowski Iamurri, Claudio Cerchione, Nicola Gentili, Valentina Danesi, Mattia Altini, Giovanni Paganelli, Domenico Barone

**Affiliations:** 1 Radiology Unit, Istituto Scientifico Romagnolo per lo Studio e la Cura dei Tumori (IRST) IRCCS, Meldola, Italy; 2 Hematology Unit, Istituto Scientifico Romagnolo per lo Studio e la Cura dei Tumori (IRST) IRCCS, Meldola, Italy; 3 Healthcare Administration, Istituto Scientifico Romagnolo per lo Studio e la Cura dei Tumori (IRST) IRCCS, Meldola, Italy; 4 Nuclear Medicine Unit, Istituto Scientifico Romagnolo per lo Studio e la Cura dei Tumori (IRST) IRCCS, Meldola, Italy

**Keywords:** COVID-19, Radiology imaging, Imaging management, Health emergency

## Abstract

In Italy, the first case of the coronavirus disease 2019 (COVID-19) was officially reported on 20.02.2020. The disease has since rapidly evolved, causing a public health emergency throughout the country but especially in our region, one of the most widely affected areas. We reorganized the daily routine of our cancer center to reduce the risk of contagion. A temporary tensile structure was set up as an entry-point triage, and a COVID-19 route was created with a dedicated CT scanner. A pre-access telephonic triage was performed the day before a patient was scheduled to come in for an examination. At the time of writing (May 4), 4053 patients had been to our center since the emergency officially began (9.03.2020) and the COVID-19 route had been activated for only 9 paucisymptomatic outpatients and 7 symptomatic inpatients. We also re-evaluated patient radiology examination lists and rescheduled non-urgent tests in consensus with the referring oncologist. Out of a total of 1438 patients scheduled for radiological examinations, 456 were postponed for a total volume reduction of 29.1%. Nine asymptomatic patients with typical CT findings of COVID-19 were identified during routine CTs, but none were RT-PCR-positive for SARS-CoV-2. We guaranteed all urgent and semi-urgent examinations, including those to stage newly diagnosed cancers and to evaluate response to treatment, ensuring the continuation of the diagnostic and therapeutic pathway of our patients. The measures we took were instrumental in keeping the institute COVID-19-free. We also describe the planned measures to resume normal clinical practice at the center.

## Background

The first case of the coronavirus disease 2019 (COVID-19) in Italy was reported in a town in the north on February 20, 2020. Since then, the disease has rapidly evolved, causing a public health emergency, especially in our region, one of the three most widely affected areas of the country. As of the writing of this paper (May 4), there have been a total of 210,717 cases of COVID-19 in Italy (26,016 in our region) and 28,884 deaths (median age 79 years, males 63.3%), with an Italy case fatality rate of 13.7% [1]. Given the lack of widespread testing of asymptomatic and paucisymptomatic patients, these numbers almost certainly underestimate the extent of the “severe acute respiratory syndrome coronavirus 2” (SARS-CoV-2) infection and overestimate the case fatality rate [2].

Patients with cancer, hematological malignancies and other diseases associated with immunosuppression are considered high-risk individuals. Although data are currently somewhat limited, immunocompromised patients appear to have a higher risk of contracting the infection and also of developing a more severe trend [3–5]. Within this scenario, we reorganized the daily routine of our Radiology Unit to reduce the risk of contagion among fragile patients, with many imaging procedures temporarily shut down or scaled down as the pandemic continued. The present study describes our daily routine imaging activity for the management of cancer patients during the SARS-CoV-2 pandemic, which contributed substantially to keeping our institute COVID-19-free.

## Measures adopted in our cancer center

Our institute covers a catchment area of over 1,100,000 inhabitants and serves more than 25,000 patients/year. The Radiology Unit performs examinations on suspect or confirmed cancer patients referred to our institute after oncological or hematological evaluation.

Although national lockdown measures in Italy were announced March 9, 2020, our regional government issued COVID-19 guidelines in late February to permit the organization of first-level dedicated triage for patients with respiratory symptoms, integrated with second-level triage including chest X-ray (CXR), computed tomography (CT) and reverse transcription-polymerase chain reaction (RT-PCR) testing. Based on these guidelines, our Healthcare Administration decided to adopt several structural measures to reduce the risk of contagion.

## Evaluation of examination list and schedules

Following national and international recommendations [4–6] and in agreement with Healthcare Administration and oncologist colleagues, we decided to:

- proceed with all clinically urgent or semi-urgent examinations, including staging for new cancer diagnosis, evaluation of response to therapy (both for patients taking part in clinical trials and those undergoing standard chemotherapy and/or radiotherapy) and follow-up imaging of high-risk cancers;
- reschedule all non-urgent examinations (4- 6- and 12-month follow-up for low-risk cancer).

In this setting, a radiologist checked patient lists the day before a CT, magnetic resonance imaging (MRI), X-ray and ultrasound (US), confirming or postponing the examination in consensus with the referring oncologist.

## Pre-access telephonic triage

Patients with a confirmed examination at the center then underwent a telephonic triage staffed by diagnostic radiographers, nurses or physicians to rule out the presence of flu-like symptoms and potential contact with verified or suspected COVID-19 cases in the previous 14 days. Results of the telephonic triage were inserted into a specifically developed software that synchronized all information. Patients with symptoms suspicious of COVID-19 were invited to contact their general practitioner, and the examination was rescheduled once the symptoms had resolved and the SARS-CoV-2 infection had been ruled out. Hospital staff also performed a pre-access daily triage and were required to stay at home if they had any flu-like symptoms or had been in recent contact with a person with likely or confirmed COVID-19, until a RT-PCR nasopharyngeal specimen had been performed and was negative.

## Tensile structure triage as entry filter

On March 16, a tensile structure was erected by the Civil Defense Corps at the entrance to our center to serve as a regular filter for patients and medical staff entering the hospital. A daily pre-entrance triage (first step) was performed by the institute’s nurses, after which members of the Civil Defense Corps directed the flow of patients, ensuring that social distancing measures were respected.

## *COVID-19 route* and dedicated CT scanner

A patient with suspected COVID-19 was transferred from the tensile structure to the Emergency Room via a special second entrance for medical evaluation. If the suspicious of COVID-19 was confirmed, the physician alerted the Radiology Unit and the patient underwent a chest CT. In an effort to keep the institute COVID-19-free, a special *COVID-19 route* was set up with a dedicated CT scanner (General Electric, Discovery 16 slice CT scanner) situated in an area with an entrance and exit separate to those of other patients requiring radiological examinations. A chest CT is considered a first-line imaging modality in highly suspicious cases of COVID-19 because it guarantees the rapid management of symptomatic patients arriving at the hospital. Although RT-PCR testing is considered the reference standard for the diagnosis of COVID-19 (despite having only moderately high sensitivity, i.e., 66–80%) [7], our daily clinical practice soon highlighted the problem of having to wait > 24 h for the results of nasopharyngeal swabs. Some studies have reported a higher sensitivity for CT than for RT-PCR testing [7], with lung parenchymal changes detected by the former in up to 54% of asymptomatic patients, showing a predominance of ground-glass opacity [8]. CT scans can also suggest alternative diagnoses to COVID-19 [9, 10]. However, CT should not be used for screening or as a first-line diagnostic test for COVID-19 but rather as an interim measure, and even then with due caution until more widespread testing is available. Overall, CTs in this setting can be used to inform decisions whether to test for COVID-19, admit patients to hospital or start other treatments [11–13].

This is because even though CT abnormality patterns for the diagnosis of COVID-19 are well known and classified as typical, indeterminate or atypical [14], they are not specific for COVID-19 and overlap with other infections (including influenza H1N1, SARS and MERS), with pulmonary toxicity induced mainly by chemotherapy and immunotherapy [15, 16], and with disease progression, especially in lung cancer patients [17].

At our center, the dedicated CT scanner is reserved for suspected COVID-19 cases identified during the tensile structure triage and for suspected COVID-19 inpatient cases.

As a precaution, we decided to perform nasopharyngeal RT-PCR testing for all patients with CT findings suspicious for COVID-19 (typical, indeterminate or atypical features). If a CT scan showed that symptoms were related to a condition other than COVID-19 (such as lung cancer progression), the patient continued his diagnostic or therapeutic pathway in the center. The same CT was also used for inpatients (all screened by RT-PCR pre-hospitalization and only admitted in the event of a negative result) with suspected COVID-19 to keep the other part of the Radiology Unit COVID-free and guarantee that non-delayable CT examinations could be carried out.

## General measures taken in the Radiology Unit [18–20]

- A distance of at least 2 m between patients is maintained in waiting rooms to respect social distancing, with a maximum of three people per waiting room;
- Radiology Unit staff wear gloves, a hair cap, surgical mask and daily scrubs. They are required to wash their hands frequently and to disinfect their workstation. Radiologists who perform US must also wear a face shield or goggles. Additional PPE (FFP3 mask, goggles or face shield, disposable gown and socks) is used when dealing with patients with symptomatic suspected COVID-19;
- Patient table and chair, CT and MRI gantry and US probe are always cleaned after each examination. The assigned team disinfects the room (including the computer keyboard, mouse and desk used by the radiographer) after an examination has been carried out on a symptomatic suspected case of COVID-19;
- Indoor spaces are frequently ventilated and cleaned on a daily basis [21, 22].

## Radiology Unit data

We decided to evaluate our data over an 8-week period during the Italian lockdown (March 9—May 3, 2020) to see whether our management strategy could be considered safe for cancer patients.

During the study period, 4053 patients (for a total 17,793 accesses) came to our institute for diagnostic or therapeutic purposes, of whom 942 (for a total of 1110 accesses) underwent at least one imaging examination. The *COVID-19 route* was only activated for 9 paucisymptomatic patients identified at the tensile structure triage for whom CT revealed one case of typical COVID-19 pattern (RT-PCR negative for SARS-CoV-2 and final diagnosis of H1N1-related pneumonia), one case of indeterminate COVID-19 pattern (RT-PCR negative for SARS-CoV-2 and final diagnosis of bacterial pneumonia) and 7 cases of COVID-19-negative CT (2 negative CTs and 5 cases of cancer progression). In the 2 negative CTs, RT-PCR was performed but proved negative.

The COVID-19-dedicated CT scanner was also used 7 times for inpatients who became symptomatic after admission. Of these, 2 patients showed an atypical COVID-19 pattern, one with a final diagnosis of pneumococcal pneumonia and the other with *pneumocystis jirovecii* pneumonia, both confirmed by bronchoalveolar lavage. In 3 patients, CT did not reveal any COVID-19-suspected findings; 2 were diagnosed with bacterial pneumonia and one with a biliary drainage infection.

In 2 inpatients, CT scans were negative for pneumonia, but a final diagnosis of COVID-19 was made after performing a new RT-PCR test. The patients, both immunocompromised, were subsequently transferred to the nearest COVID-19 hospital.

The Radiology Unit reviewed 1438 outpatients scheduled for radiological examinations and performed as many triage phone calls. Four hundred and fifty-six appointments were rescheduled (for a total radiology volume reduction of 29.1%), with 26 patients refusing the new appointment because they were afraid of leaving home and getting COVID-19. Four hundred and eighteen non-urgent appointments were rescheduled, and only 12 were postponed because the patients had flu-like symptoms (0.83% of the total number of appointments and 2.36% of rescheduled patients). There were no cases of delayed examinations because a patient reported being in contact with someone with confirmed COVID-19. During the study period, we performed imaging examinations on 128 inpatients.

**Fig. 1:**
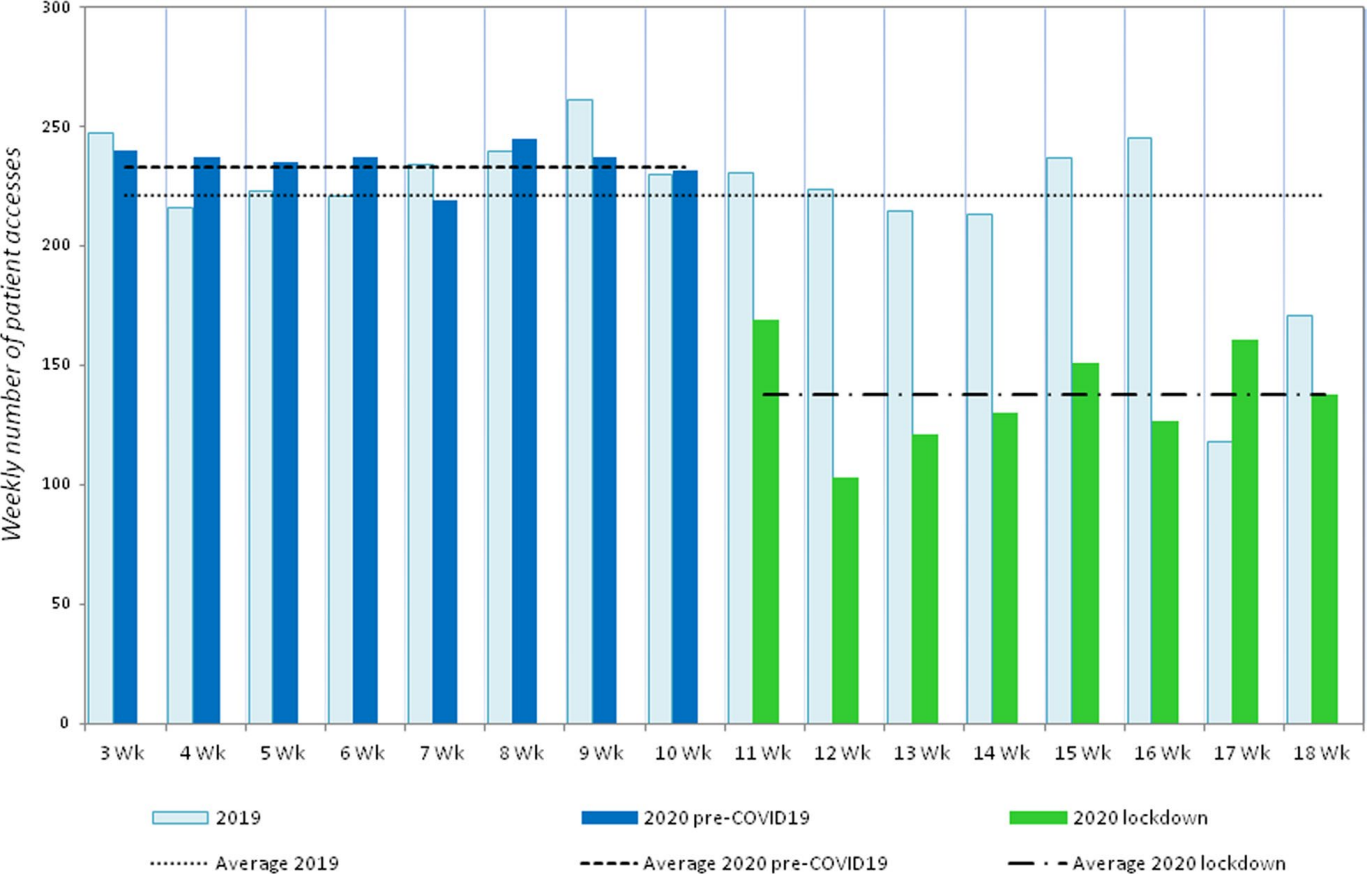
The bar chart compares the weekly numbers of patient accesses to the Radiology Unit of our institute for imaging examinations from week 3 to week 18 of 2019 and 2020. Both inpatient and outpatient accesses were considered. A considerable reduction in the number of patient accesses was registered during the lockdown (green bars), which occurred from week 11 to week 18, with respect to the same period in 2019. The decreased access resulted in a reduction in overall radiology activities. N.B. Week 17 of 2019 only had 3 working days because of a national holiday

A comparison between data collected in 2020 before and during the lockdown was made with data collected in the same period in 2019. Focusing on the period before lockdown, data were comparable between the two years. The maximum reduction in patient access to the Radiology Unit was registered during the second and third weeks of lockdown in March (Fig. 1). In contrast, the following few weeks revealed a gradual increase in patient accesses week after week, probably as a consequence of the progressive adjustment of procedures.

With regard to imaging modalities performed in 2020 compared to the same period in 2019 (from 09.03.2019 to 03.05.2019), data gathered by RIS/PACS showed a reduction in volume of 26.0% for CT, 34.7% for MRI, 34.8% for X-ray and 58.5% for US.

**Fig. 2:**
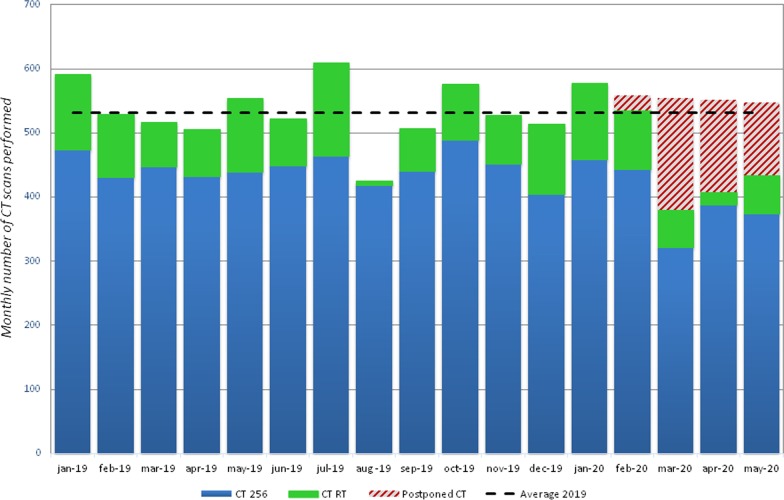
The bar chart represents the monthly number of CT examinations performed from January 2019 to May 2020 for the two CTs available in our facility (CT 256 and CT RT). The black dashed line refers to the average data for 2019. The black dashed line represents the rescheduled CTs from the end of February 2020 and during lockdown with respect to the originally planned examinations

Given that CT is the main technique used to assess disease response and progression in cancer patients, CT activity was subject to lesser reduction than other techniques. In particular, the maximum reduction in the number of CTs performed was registered in the first month of the lockdown in March (Fig. 2). The number of CT scans performed that month was 29% lower than the average number performed in 2019, while in April and May, the reduction was 23% and 18%, respectively.

Our data are better than expected, especially if we consider that Cavallo et al. recently estimated that radiology practices would see a decrease in imaging volumes of between 50 and 70% for at least 3 months [23]. Moreover, www.quinsite.com reported that average daily routine radiology volumes in the USA were down 50.9% in what it called week 6 of the COVID-19 outbreak compared with before the start of the survey period on March 16. Quinsite also measured the impact of the COVID-19 pandemic by imaging modality: MRI -47.1%, US -44.2%, X-ray -43.1% and CT -35.9%.

During this period, we incidentally identified 9 patients with typical findings of COVID-19 in asymptomatic patients during routine CT scans. In accordance with Fleischner Society recommendations and our own Health Administration procedures, we carried out RT-PCR testing in these patients to identify potentially occult infections and limit further transmission both within the community and in the hospital [11]. None of the patients were RT-PCR-positive; there were 6 cases of pulmonary drug toxicity (5 immunotherapy-induced pneumonitis and one of interstitial pneumonia induced by bleomycin), 2 cases of disease progression and one case of influenza. The CT room was thoroughly disinfected after each of the scans. These data highlight the difficulty in correctly interpreting overlapping patterns between COVID-19 pneumonia and drug-induced pneumonia or disease progression, especially lung cancer. At the time of the writing of this manuscript, there had been no cases of COVID-19 among the staff of the Radiology Unit.

## Future prospects

There are now plans in Italy to slowly reduce healthcare lockdown measures and restart non-urgent care in areas with a low incidence of COVID-19, similar to the “Opening up America again” guidelines published in April 19 [24].

Following the indications of our Regional government, we will slowly reopen imaging facilities while also maintaining the safety measures successfully used for patients and staff during the lockdown. The expected surge of examinations will be managed by:

- extending the operating hours of US and MRI and using another CT scan normally used by the Radiotherapy Unit for CT setup (Philips Brilliance Big Bore 16-slice CT scanner);
- developing shorter imaging protocols for MRI examinations;
- carefully evaluating with our oncologist colleagues the need to reschedule all follow-up examinations canceled;
- modifying the setup of the triage tensile structure to streamline access to the center for patients and healthcare operators, given that it will be in use for at least 12 months.

## Conclusions

The general measures adopted at our cancer center were substantially in line with what has been done in “Cancer Core Europe” [25], but maybe not completely applicable to general hospitals.

After 8 weeks of triage and decreased activities, it can be concluded that our Radiology Unit’s management of patients proved safe both for patients and staff. In fact, our daily routine contributed substantially to keeping our institute and radiology staff COVID-19-free, despite the fact that we are situated in one of the three worst hit COVID-19 areas in Italy. In accordance with the mission of healthcare and research of our institute, we guaranteed all urgent and semi-urgent examinations, including those to stage newly discovered cancer and those to assess response to therapy (i.e., patients taking part in clinical trials and also those undergoing standard chemotherapy and/or radiotherapy, which accounts for around 200 patients/day), thus ensuring the continuity of the diagnostic and therapeutic pathway of cancer patients attending our center. Our policy was substantially in line with what has currently been published [2].

Radiologists need to become familiar with COVID-19 CT patterns so that they can identify the infection in patients imaged for other reasons and refer them for RT-PCR testing. Our recent experience highlighted the difficulty in distinguishing between COVID-19 patterns and drug-induced pneumonitis (especially immunotherapy) or progression of lung cancer because of overlapping radiological features. This may be a less frequent occurrence outside of cancer institutes where the pretest probability for COVID-19 for non-oncological patients with symptoms (according to Bayesian logic) is very high, but obviously, it is pertinent to our daily clinical practice. Thanks to the precious collaboration between the staff of our institute (oncologists, healthcare management, nurses, diagnostic radiographers) and the Civil Defense Corps, we are ready to slowly move into the next phase of the pandemic, “Opening up Cancer Imaging again,” keeping people safe.

## Data Availability

The datasets generated and/or analyzed during the current study are available from the corresponding author on reasonable request.

## Abbreviations

COVID-19: Coronavirus disease 2019
CRX: Chest X-ray
CT: Computed tomography
MERS: Middle east respiratory syndrome
MRI: Magnetic resonance imaging
RT-PCR: Reverse transcription-polymerase chain reaction
SARS: Severe acute respiratory syndrome
SARS-CoV-2: Severe acute respiratory syndrome coronavirus 2
US: Ultrasound
